# Thrombolysis for ECMO oxygenator thrombosis

**DOI:** 10.1186/s13054-023-04433-6

**Published:** 2023-04-14

**Authors:** Fabio Silvio Taccone, Leda Nobile, Filippo Annoni

**Affiliations:** Department of Intensive Care, Hôpital Universitaire de Bruxelles (HUB), Erasme Hospital, Université Libre de Bruxelles (ULB), Route de Lennik, 808, 1070 Brussels, Belgium

Although uncommon, oxygenator thrombosis in extracorporeal membrane oxygenation (ECMO) devices remains a potential reason for exchanging the circuit, the risk of thrombosis increases with the duration of ECMO therapy and with procoagulant conditions, such as COVID-19 [1, 2]. With a worldwide increased demand for ECMO support, alternative methods to prolong lifespan of the oxygenator have been considered. Recombinant tissue-type plasminogen activator (rtPA) could be a potential approach in this setting [3].

From March 1, 2020, to March 30, 2022, 3 patients from a population of 99 were treated with either veno-arterial (V-A) and veno-venous (V-V) ECMO or extra-corporeal CO_2_ removal (ECCO_2_R) support in our Intensive Care Unit (ICU) required the injection of rtPA for acute oxygenator thrombosis. Characteristics of the study cohort are reported in Table 1. Informed consent was waived by our Ethics Committee because the data collection was retrospective from the data patient monitoring system (PDMS). The decision to administer rtPA was discussed and decided in the multidisciplinary medical round.

**Table 1:** Characteristics of the study cohort

	Patient 1		Patient 2		Patient 3	
Age, years	49		61		53	
Gender	Male		Male		Male	
Underlying disease	COVID-19		Inhalation		COVID-19	
Time to ECMO*, days	3		8		5	
ECMO Configuration	VV		ECCO2R		VV	
ECMO Device	LivaNova		EUROSETS		LivaNova	
Time from ECMO* to rtPA, days	7		1		5	
Days on ECMO support, days	15		7		19	
Number of rtPA injections	2		2		2	
Type of rtPA	Alteplase		Alteplase		Alteplase	
Total dose of rtPA, mg	10		5		7.5	
Systemic anticoagulation	UFH		UFH		UFH	
Baseline target anti-Xa activity	0.2–0.4		0.3–0.5		0.2–0.4	
Anti-Xa at clotting	0.13		0.31		0.15	
New target anti-Xa activity	0.4–0.6		0.4–0.6		0.4–0.6	
Bleeding	None		None		None	
Outcome	Death		Discharged		Death	
	**Before**	**After**	**Before**	**After**	**Before**	**After**
ECMO Blood flow, L/min	3.5	5.5	0.4	0.8	2.4	4.5
Pump velocity, rpm	3200	2800	NA	NA	3300	2750
Trans-membrane Gradient, mmHg**	265	143	65	38	228	121
Arterial Oxygenation, %	55	93	95	96	71	95

*ECMO *extra-corporeal membrane oxygenation, *rtP2* recombinant tissue-type plasminogen activator, *ECCO2R* extra-corporeal CO_2_ removal, *UFH* unfractionated heparin, *NA* not available

*or ECCO_2_R

**Calculated as the difference between the pressure measured on the outflow and inflow of the oxygenator (ECMO) or shown on the device screen (ECCO2R)

The first patient was on V-V ECMO during the first COVID-19 wave and was fully dependent on the ECMO support because of severe respiratory failure; after 4 days on ECMO, the oxygenator showed rapid signs of clotting (i.e., ECMO flow reduced to 3 L/min) and arterial oxygenation dropped to 55%. Because of no chance of survival considering the time required to have a perfusionist available, the administration of low-dose rtPA (5 mg bolus followed by additional 5 mg after 5 min) into the drainage section of the oxygenator was decided; within 15 min, the ECMO flow returned to the initial baseline values. Anticoagulation was adjusted to higher targets and no recurrence of oxygenator clotting was observed thereafter. The second patient presented severe respiratory acidemia (i.e., pH 7.18; PaCO2 83 mmHg) with protective lung ventilation requiring ECCO2R; within 3 h from onset of therapy, oxygenator thrombosis occurred in non-working hours and it was decided to administer rtPA to avoid complete circuit and cannula clotting. Drug administration helped to dissolve the clot and result in a normal function of the device. Similar results were observed in the third patient; no hemorrhagic complication was observed within the 5 days following rtPA injection. Heparin was not interrupted during thrombolysis.

This is the second report on the use of “off-label” rtPA for oxygenator thrombosis in ECMO patients. In another small cohort, 4 patients were successfully treated with several administrations of rtPA [3], without significant complications; however, all patients had only moderately impaired systemic oxygenation, not justifying immediate oxygenator exchange, while in our cohort two patients had life-threatening respiratory compromise with severe hypoxemia. Unfractionated heparin (UFH) remains the anticoagulant of choice for patients on ECMO, although the optimal anticoagulation target and the most effective coagulation parameter to use to adjust heparin regimens remain unknown in these patients. The reason we decided to continue with UFH was due to the relatively low anti-Xa values at clotting, which suggest a potentially insufficient anticoagulation regimen rather than the ineffectiveness of UFH. Moreover, the indication for oxygenator exchange in case of suspected thrombosis is also undetermined and might be based on membrane performance, hemolysis and/or patient’s status [4]. Further studies are required to understand the timing, dose and potential complications of rtPA use for acute oxygenator thrombosis in ECMO patients. Our findings suggest that rtPA given at lower doses than thrombolysis for pulmonary embolism or ischemic stroke (i.e., 5–20 mg) could be effective to treat life-threatening oxygenator thrombosis.

## Data Availability

On request to the corresponding author.
